# Low global sensitivity of metabolic rate to temperature in calcified marine invertebrates

**DOI:** 10.1007/s00442-013-2767-8

**Published:** 2013-09-14

**Authors:** Sue-Ann Watson, Simon A. Morley, Amanda E. Bates, Melody S. Clark, Robert W. Day, Miles Lamare, Stephanie M. Martin, Paul C. Southgate, Koh Siang Tan, Paul A. Tyler, Lloyd S. Peck

**Affiliations:** 1 School of Ocean and Earth Science, University of Southampton, National Oceanography Centre Southampton, European Way, Southampton, SO14 3ZH UK; 2 British Antarctic Survey, Natural Environment Research Council, High Cross, Madingley Road, Cambridge, CB3 0ET UK; 3 Institute of Marine and Antarctic Studies, University of Tasmania, Hobart, TAS Australia; 4 Zoology Department, University of Melbourne, Parkville, 3010 Australia; 5 Department of Marine Science, University of Otago, Dunedin, New Zealand; 6 14 Duck Lane, Eynesbury, St. Neots, Cambridgeshire PE19 2DD UK; 7 Centre for Sustainable Tropical Fisheries and Aquaculture, School of Marine and Tropical Biology, James Cook University, Townsville, QLD 4811 Australia; 8 Tropical Marine Science Institute, National University of Singapore, 14 Kent Ridge Road, Singapore, 119223 Singapore; 9 Present Address: Australian Research Council Centre of Excellence for Coral Reef Studies and School of Marine and Tropical Biology, James Cook University, Townsville, QLD 4811 Australia

**Keywords:** Oxygen, Latitude, Activation energy, Energetics, Climate change

## Abstract

Metabolic rate is a key component of energy budgets that scales with body size and varies with large-scale environmental geographical patterns. Here we conduct an analysis of standard metabolic rates (SMR) of marine ectotherms across a 70° latitudinal gradient in both hemispheres that spanned collection temperatures of 0–30 °C. To account for latitudinal differences in the size and skeletal composition between species, SMR was mass normalized to that of a standard-sized (223 mg) ash-free dry mass individual. SMR was measured for 17 species of calcified invertebrates (bivalves, gastropods, urchins and brachiopods), using a single consistent methodology, including 11 species whose SMR was described for the first time. SMR of 15 out of 17 species had a mass-scaling exponent between 2/3 and 1, with no greater support for a 3/4 rather than a 2/3 scaling exponent. After accounting for taxonomy and variability in parameter estimates among species using variance-weighted linear mixed effects modelling, temperature sensitivity of SMR had an activation energy (*E*a) of 0.16 for both Northern and Southern Hemisphere species which was lower than predicted under the metabolic theory of ecology (*E*a 0.2–1.2 eV). Northern Hemisphere species, however, had a higher SMR at each habitat temperature, but a lower mass-scaling exponent relative to SMR. Evolutionary trade-offs that may be driving differences in metabolic rate (such as metabolic cold adaptation of Northern Hemisphere species) will have important impacts on species abilities to respond to changing environments.

**Electronic supplementary material:**

The online version of this article (doi:10.1007/s00442-013-2767-8) contains supplementary material, which is available to authorized users.

## Introduction

The physiological plasticity of ectotherms is correlated with large-scale geographic patterns in environmental conditions (e.g. Gaston et al. 2009). Gaining a mechanistic understanding of how thermal performance of organisms varies with latitudinal range is, therefore, a key tool for understanding how species distributions are likely to be affected by changing climate (Pennisi 2005). As temperature is arguably the most important environmental variable driving the physiological rates of ectotherms (Hochachka and Somero 2002), geographic patterns of physiological adaptation are predicted to be primary correlates of an environmental thermal regime and thus reflected in species’ distributions (e.g. Addo-Bediako et al. 2000). Metabolic rate is a key component of energy budgets and investigations into its scaling with morphology and temperature have been extensive (Heusner 1985; Bergmann 1847), with global comparisons being used to identify geographic patterns of metabolic rate and potential mechanisms underlying these patterns (e.g. Clarke and Johnston 1999).

**Table 1:** Collection site, season and environmental temperature (at time of collection) for each species

Location	GPS coordinates	Species	Latitudinal range^a^	Water temperature (°C) (dew point ± 1 SD)	*n*	Season
Northern Hemisphere, tropical						
Laternulid bivalves						
Straits of Johor, Singapore	1.444°N, 103.741°E	*Laternula truncata,*	42S–12S	29.9 (±0.5)	29	Aseasonal
		*Laternula boschasina*	1N–35N		19	
Southern Hemisphere, tropical						
Buccinid gastropods						
Lucinda, QLD, Australia	18.531°S, 146.341°E	*Phos senticosus*	43S–37N	28.3 (±0.1)	30	Aseasonal
Townsville, QLD, Australia	19.240°S, 146.796°E	*Cantharus fumosus*	31S–18N	28.3 (±0.1)	31	Aseasonal
Southern Hemisphere, temperate						
Laternulid bivalves						
Port Phillip Bay and Barwon Heads, VIC, Australia	38.329°S, 144.600°E	*Laternula recta*	43S–20S	14.2 (±0.1)	13	Winter
Buccinid gastropods						
Port Phillip Bay and Barwon Heads, VIC, Australia	38.329°S, 144.600°E	*Cominella lineolata*	43S–25S	14.2 (±0.1)	21	Winter
Brachiopods						
Doubtful Sound, New Zealand	45.314°S, 166.987°E	*Liothyrella neozelanica,*	54S–32S	14.1 (±0.2)	29	Summer
		*Terebratella sanguinea,*	54S–32S		26	
		*Notosaria nigricans*	54S–32S		16	
Northern Hemisphere, temperate						
Echinoids						
Torquay, UK	50.458°N, 3.533°W	*Psammechinus miliaris*	47N–61N	18.9 (±0.4)	33	Summer
Buccinid gastropods						
Southampton, UK	50.878°N, 1.384°W	*Buccinum undatum*	36N–79N	18.9 (±0.4)	28	Summer
Southern Hemisphere, polar						
Laternulid bivalves						
Rothera Research Station, Adelaide Island, Antarctica	67.578°S, 68.165°W	*Laternula elliptica,*	78S–53S	0.7 (±0.2)	32	Summer
Buccinid gastropods						
Rothera Research Station, Adelaide Island, Antarctica	67.578°S, 68.165°W	*Neobuccinum eatoni*	48S–78S	0.7 (±0.2)	29	Summer
Echinoids						
Rothera Research Station, Adelaide Island, Antarctica	67.578°S, 68.165°W	*Sterechinus neumayeri*	78S–54S	0.7 (±0.2)	25	Summer
Brachiopods						
Rothera Research Station, Adelaide Island, Antarctica	67.578°S, 68.165°W	*Liothyrella uva*	78S–46S	0.7 (±0.2)	28	Summer
Northern Hemisphere, polar						
Buccinid gastropods						
Ny Ålesund, Svalbard, Arctic	78.956°N, 11.970°E	*Buccinum glaciale*	42N–79N	4.3 (±0.4)	12	Summer
		*Buccinum* cf. *groenlandicum*	68N–79N		21	

Data sorted from low to high latitudes

^a^Distributions from the Global Biodiversity Information Facility

Significant differences in the scaling of metabolic rate with body mass are known across morphologically distinct marine taxa, resulting in scaling exponents largely between 2/3 and 1 (Glazier 2010). Modular organisms, such as clonal bryozoans, can have a mass-scaling exponent for metabolic rate that is close to unity (*M* ^1^; Hughes and Hughes 1986; Peck and Barnes 2004). Euclidean geometry of surface area to volume predicts a 2/3 mass-scaling exponent in most unitary organisms (e.g. Glazier 2010). However, scaling relationships are also modified by the balance between energy supply and demand, which are in turn related to temperature. Even unitary organisms whose metabolic rate is directly proportional to the energy requirements of activity and minimal tissue maintenance should have a mass-scaling exponent close to 1, whereas organisms whose metabolic rate is restricted by the flux of resources across surfaces should have a 2/3 mass-scaling exponent (Glazier 2010). The balance between energy requirements and surface area restrictions are thought to determine where the metabolic scaling exponent falls between these two metabolic-level boundaries (MLB hypothesis; Glazier 2010). A number of other restrictions on the allometry of metabolic rates, such as the evolutionary diversification of cell and genome size (which also predicts a scaling exponent between 2/3 and 1) are also thought to add to the variation of scaling exponents (Kozlowski et al. 2003).

By contrast, there is a long-held assumption that the mass-scaling exponent should be restricted to 3/4 (Brody and Proctor 1932; Brody 1945; Kleiber 1947), which is a central tenet of the metabolic theory of ecology (MTE). The MTE is a resource network model proposing that the fractal-like design of exchange surfaces and distribution networks results in a universal 3/4 power scaling relationship between standard metabolic rate (SMR) and body mass (Banaver et al. 2002). The MTE (Gillooly et al. 2001) has reignited the debate over whether there is a universal scaling exponent and the significance of any departures from 3/4 (e.g. Clarke 2006; Gillooly et al. 2006).

The MTE is also underpinned by the thermodynamic reaction kinetics of physiological reactions which have an average activation energy (*E*_a_) of 0.65 eV (Gillooly et al. 2001). Whilst many forms of life, from unicellular organisms to ecosystems, fit with the concepts of the MTE (Gillooly et al. 2001, 2006; Brown et al. 2004; Allen et al. 2007; Perkins et al. 2012), the value of *E*_a_ varies consistently due to specific traits, such as body size and morphology, and thus between taxa, trophic groups and habitats (Dell et al. 2011; Huey and Kingsolver 2011). There are also some notable counter arguments and exceptions that challenge its universality (Clarke 2004; Clarke and Fraser 2004; O’Connor et al. 2007; Terblanche et al. 2007; Irlich et al. 2009; Marshall and McQuaid 2011) with non-climatic thermal adaptation (Marshall and McQuaid 2011), the evolutionary trade-off hypothesis (Clarke 2006; Clarke and Fraser 2004) and metabolic cold adaptation (Krogh 1914; Scholander et al. 1953; White et al. 2012) all proposed as evolutionary adjustments that would cause metabolic rate to depart from the principles of the MTE.

Whilst wide-scale analyses of ectotherm metabolic rate across latitudes, from the tropics to the poles, have been conducted for perciform fish (Clarke and Johnston 1999) and bivalve molluscs (Peck and Conway 2000), these studies compiled data collected using a variety of experimental protocols. To our knowledge, there are no reported studies that have analysed ectotherm metabolic rate from the tropics to the poles using a single, consistent, experimental methodology. We measured the SMR of calcifying marine invertebrate species that occur at temperatures from 0 to 30 °C across a 70° latitudinal gradient in both hemispheres, to test whether there was greater evidence for a 2/3 or 3/4 mass-scaling exponent of SMR, or whether they varied between the metabolic level boundaries of 2/3 and 1. *E*a was expected to change with a consistent temperature coefficient (*Q*_10_) of 2–3 (Clarke 1983), giving an Arrhenius slope of −7.40 K, which is equivalent to an *E*_a_ = 0.65 eV, across taxa. Our results provide strong global experimental support that SMR is shaped by environmental thermal regime over evolutionary timescales and indicates differences between hemispheres that need to be considered during energy budget modelling and meta-analyses.

## Materials and methods

We measured the SMR of 17 marine species from tropical to polar latitudes (0–30 °C) in both hemispheres. These comprised relatives from within four taxonomic groups of calcified marine invertebrates (laternulid bivalves, buccinid gastropods, echinoid urchins and rhynchonellid brachiopods), including 11 species whose oxygen consumption was measured for the first time. These species were chosen as they represent four widely distributed taxonomic groups, allowing the analyses to be constrained to benthic animals, all of which have relatively low rates of activity. Reducing potential ecological effects, such as differences in activity (Glazier 2010), can reduce many of the factors that affect SMR and are likely to influence comparisons between more distantly related species (Clarke and Fraser 2004; Rastrick and Whiteley 2011). Calcified taxa were chosen as shells act as a barrier to diffusion pathways, potentially altering the surface area to volume ratio, altering metabolic scaling exponents. The analysis therefore used ash-free dry mass (AFDM) of each species as it allowed for groups with different skeletal compositions to be compared. We further accounted for non-random sampling within these phyla by including family and species identity as a random effect (see further details below).

Marine invertebrates were collected across a wide range of latitudes: (1) low latitude—Straits of Johor, Singapore; Lucinda and Townsville, QLD, Australia; (2) mid latitude—Port Phillip Bay, Barwon Heads, Victoria, Australia; Doubtful Sound, New Zealand; Southampton and Torquay, England; and (3) high latitude—Rothera Research Station, Adelaide Island, Antarctica and Svalbard, in the Norwegian Arctic (Table 1).

All studies outside of the tropics, and with the exception of Melbourne, were conducted in summer. Individuals from the largest possible adult size range were collected for each species at each site. However, the available size range of some species, particularly some of the smaller tropical species, was limited. Shelled invertebrates were gently cleaned with a soft brush to remove any epibionts or adherent sediment. Animals were housed in aquaria to recover from the stress of collection, transportation and cleaning, and were fasted according to the approximate duration of their post-prandial rise in metabolism or specific dynamic action (SDA) at their habitat temperature. At 0 °C the SDA of Antarctic marine ectotherms often last 10–14 days (Peck 1998). The SDA for mid latitude species is expected to last 5–6 days (Peck 1998) and for lower latitudes an SDA of 1–2 days was calculated by applying a standard *Q*_10_ value of 2.5. Whilst there was some movement of animals within respirometers, the method standardized measurement of metabolic rates near maintenance levels, but not as low as long-term starved metabolic rates. In Singapore, New Zealand and Rothera, flow-through holding aquaria took filtered seawater from local bays. In Townsville, Australia, Southampton and Svalbard recirculating systems were used. Whilst no food was added during the fasting period, filter feeders likely had access to small quantities of algae and bacteria in the water.

SMR were measured using closed-system respirometry (following the methods of Morley et al. 2007a). Individuals were tested in 80- to 2,000-cm^3^ Perspex or glass respirometers, matched to the size of the organism and ambient temperature. The drop in oxygen concentration was measured using a FIBOX-3 optode system single-channel temperature-compensated oxygen meter (Pre-Sens, Regensburg, Germany) with a planar oxygen sensor glued inside each respirometer. Each oxygen sensor was calibrated every 4–5 days, to account for light attenuation and drift of individual sensors. Sensors were calibrated with a saturated seawater solution of sodium dithionite (>25 mg ml^−1^) for 0 % oxygen saturation. For calibration of 100 % oxygen saturation, seawater was vigorously aerated for 2 h, and then left to stand for 10 min to remove any super-saturation. Temperature and atmospheric pressure were recorded during both calibration and trials, and were used in calculating oxygen concentrations.

Animals were transferred, underwater, into respirometers to minimise stress and prevent the introduction of air bubbles into chambers. Before transfer, brachiopods were tapped with a mounted needle and valve closure noted to ensure they were in good physical condition. Respirometers were allowed to equilibrate in a tank of oxygen-saturated seawater, at ambient temperature before animals were placed inside and plastic mesh lids were fitted that allowed water exchange. Animals were left in these conditions overnight for ≥12 h (Peck and Conway 2000) to adjust to the respirometer before they were gently flushed with oxygen-saturated seawater and closed. Two or three control respirometers, without animals, were run to measure any background change in oxygen. All the species investigated had linear rates of oxygen consumption from 100 to <70 % oxygen saturation (data not shown). Thus experiments were run over the oxyregulating range for each species and were stopped before oxygen saturation was reduced below 80 %. Animals were then removed from the respirometer and their volume was measured using Archimedes’ principle. Seawater volume in each trial was also calculated by subtracting animal volume from the internal volume of the respirometer to calculate the amount of oxygen consumed by each animal.

Animal linear dimensions (±0.1 mm) were recorded with dial callipers and, after removing excess water, wet weight was measured (±0.001 g). Shells were dissected from tissues and both shell and tissue were dried at 60 °C to a constant weight, reweighed, and then ignited in a muffle furnace at 475 °C for 24 h. Ash remaining after ignition was placed in a desiccator to cool and AFDM was obtained by subtracting ash mass from dry mass.

The exponents from analysis of covariance models for each individual species were tested, using individual *Z*-tests, against the three competing expectations for scaling exponents of 2/3, 3/4 and 1 (Glazier 2010; Rastrick and Whiteley 2011). To account for multiple comparisons, the probabilities indicating a significant difference were Bonferroni corrected from *p* = 0.05 to 0.016 and from *p* = 0.01 to 0003.

Size had a significant effect on metabolic rate, and there were differences in slopes between species. Therefore, to investigate latitudinal patterns of metabolic rate between species of very different sizes, natural logarithm of metabolic rate for each individual was mass corrected to that of a mean-sized animal (−1.5 g natural logarithm AFDM) using the scaling exponent for all species combined (following Clarke and Johnston 1999; Gillooly et al. 2001).

We used linear modelling to compare (1) metabolic scaling exponent against the mean of mass-normalized SMR for each species (in this case the analysis was weighted by the inverse of variance), with hemisphere and temperature as fixed factors; and (2) mass-normalized SMR, for each individual, against Arrhenius temperature (1,000/K) with hemisphere and latitude as fixed factors (Arrhenius temperature was used to enable calculation of *E*_a_ and direct literature comparisons). Collinearity diagnostics (Zuur et al. 2009a) were performed by quantifying generalized variance inflation factors (GVIF) for each fixed factor and interactions with temperature or latitude (centred prior to the analysis) using the function preds.GVIF available through the R (R Development Core Team 2011) package car (Fox and Weisberg 2011). We removed interaction terms from our full model in cases where GVIF values exceeded the arbitrary threshold of 5.

Model selection consisted of first comparing generalised least squares and linear mixed effects models on the basis of *F*-tests and Akaike’s information criteria (AIC). Where it improved the model, nested taxonomy was included as a random factor (species or genus as appropriate within family within phylum). Each taxonomic level was included when it explained more than 1 % of the model variance. To identify the minimum adequate model, we included hemisphere as a fixed factor with the predictors, mass-normalized SMR and temperature, and, in a second model, Arrhenius temperature and latitude. Interactions among these factors were included in the full model and were removed following a step-wise procedure (Zuur et al. 2009b). The resulting minimum adequate model had the lowest AIC value and retained all significant random and fixed effects.

*Q*_10_ values for within-taxon comparisons were calculated using mass-normalized SMR at the temperatures recorded during the experiments following the method of Morley et al. (2007b).

## Results

### Scaling of SMR with AFDM

**Fig. 1:**
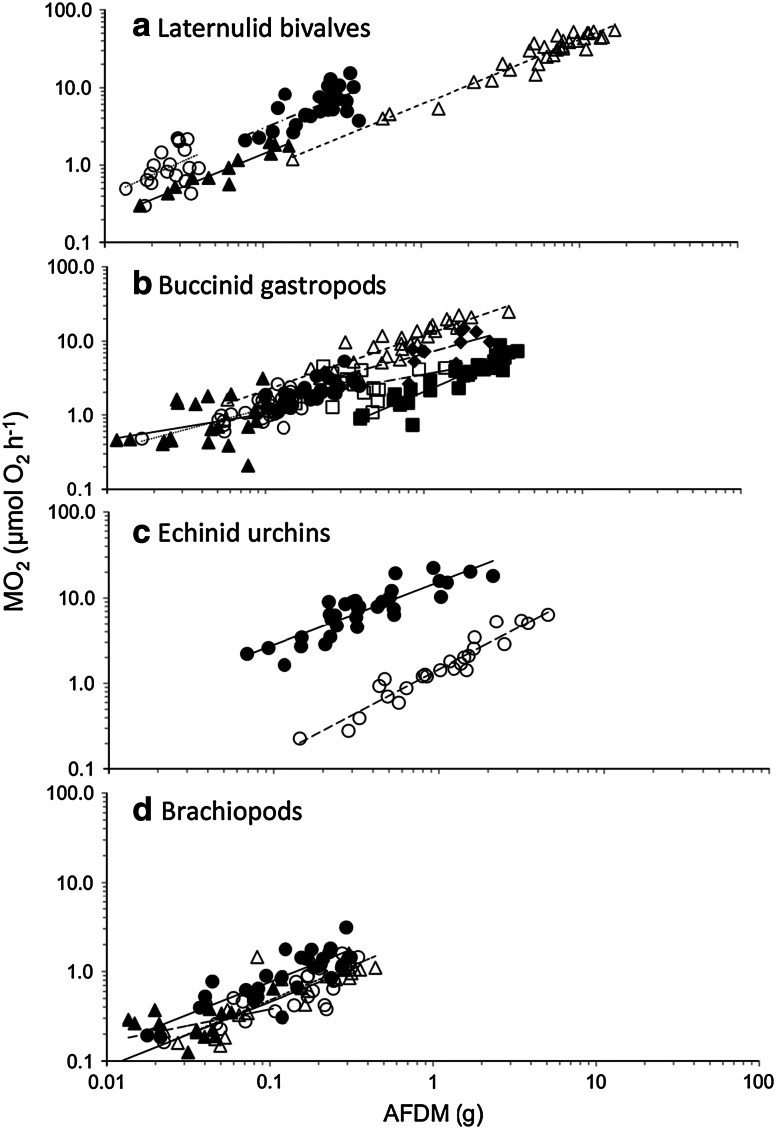
Regression fits of standard metabolic rate (SMR) (MO_2_) to ash-free dry mass (*AFDM*) for each species in the following taxonomic groups: **a** congeneric laternulid bivalves, *Laternula truncata* (*filled circles*), *Laternula boschasina* (*open circles*), *Laternula recta* (*filled triangles*), *Laternula elliptica* (*open triangles*); **b** confamilial buccinid gastropods, *Cantharus fumosus* (*filled circles*), *Phos senticosus* (*open circles*), *Cominella lineolata* (*filled triangles*), *Buccinum undatum* (*open triangles*), *Neobuccinum eotoni* (*filled squares*), *Buccinum* cf *groenlandicum* (*open squares*), *Buccinum glaciale* (*filled diamonds*); **c** echinoids from the infraorder Echinidae, *Psammechinus miliaris* (*filled circles*), *Sterechinus neumayeri* (*open circles*); and **d** brachiopods, *Liothyrella neozelanica* (*filled circles*), *Terebratella sanguinea* (*open circles*), *Notosaria nigricans* (*filled triangles*), *Liothyrella uva* (*open triangles*). Note natural logarithm scale on the axes

**Fig. 2:**
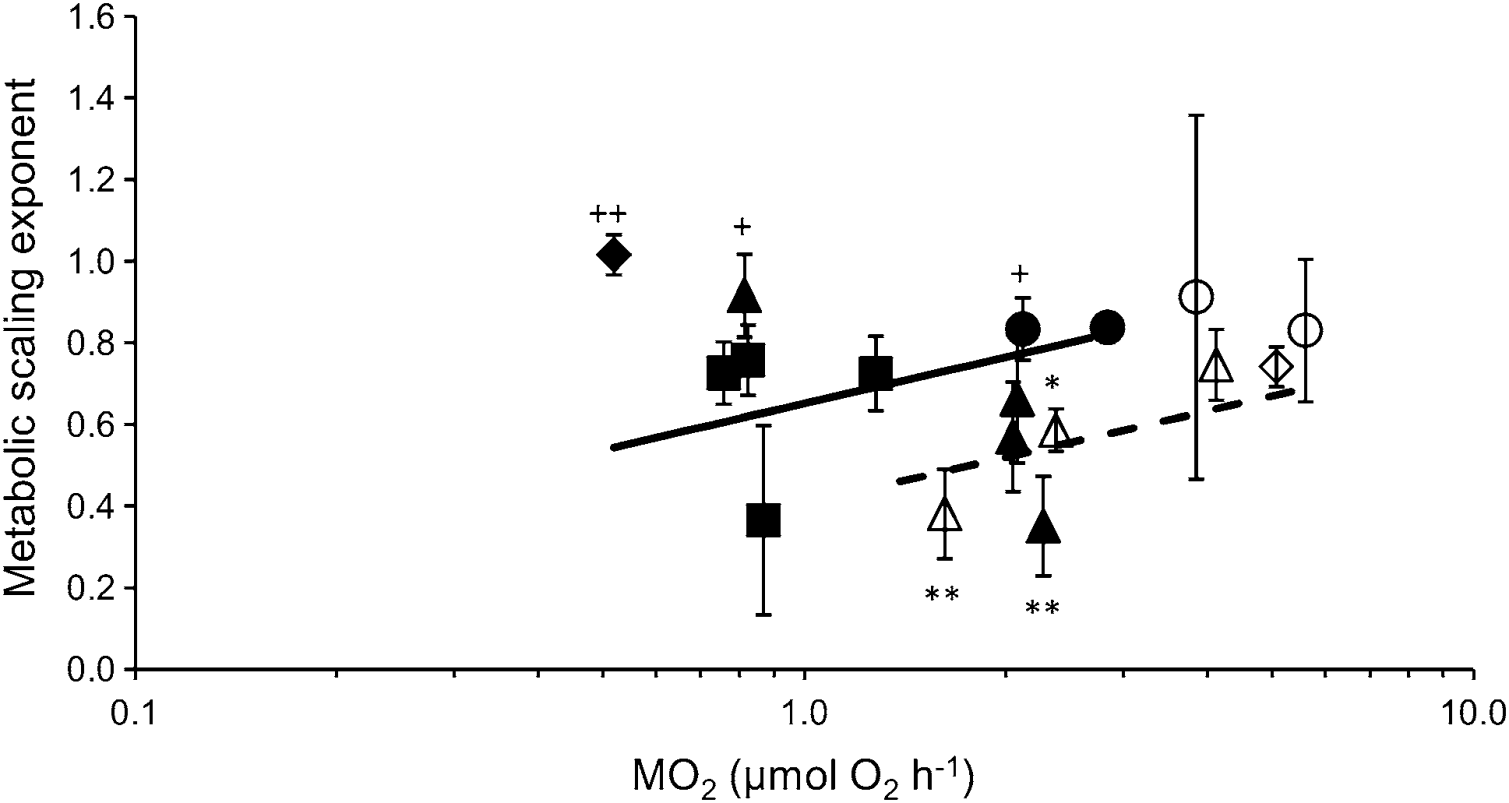
Metabolic scaling exponent against mass-normalized SMR (MO_2_ ± 1 SE) for bivalves of the genus *Laternula* (*circles*), gastropods of the family Buccinidae (*triangles*), echinoids of the infraorder Echinidae (*diamonds*), brachiopods (*squares*). *Separate lines* were fitted to Northern (*open symbols, dashed line*) and Southern (*closed symbols, solid line*) Hemisphere species for the median Arrhenius temperature using a linear model that included the inverse variance of SMR (which differed among species) as a weight. SMR was mass corrected to that of a standard-sized animal of 223 mg AFDM. Scaling exponents significantly greater than both 0.75 and 0.67 (++) or 0.67 only (+) or significantly lower than both 0.75 and 0.67 (**) or 0.75 only (*). Note natural logarithm scale on the *x-*axis

SMR scaled with size and, within each taxonomic group, larger individuals and species had a higher SMR (Fig. 1). The scaling exponent for all species combined was 0.69 ± 0.02 (*F*_1,421_ = 716.7, *p* < 0.01), which was not significantly different from 0.67 (*Z* = 0.87, *p* = 0.38). Metabolic scaling exponents of nearly all species (15 out of 17) were between 0.67 and 1.0 fitting with the expectation of the MLB hypothesis (*χ* ^2^ = 0.06, *p* = 0.80; Fig. 2; ESM Table 1). Two species (*Cominella lineolata* and *Buccinum* cf*. groenlandicum*) had metabolic scaling exponents significantly lower than both 0.67 and 0.75, while *Buccinum glaciale* had a scaling exponent lower than 0.75. *Sterechinus neumayeri* had a scaling exponent significantly higher than both 0.75 and 0.67, while two further species (*Laternula elliptica* and *Neobuccinum* eatoni) had scaling exponents significantly higher than 0.67 (ESM Table 1). These comparisons indicate no greater support for a 0.75 (*n* = 13) than a 0.67 (*n* = 12) metabolic scaling exponent (*χ* ^2^ = 0.02, *p* = 0.88).

### Metabolic scaling exponent against mass-normalized SMR

Metabolic scaling exponents increased linearly with SMR (Table 2) and were significantly related to temperature (Table 2). Overall, Southern Hemisphere species had a significantly higher scaling exponent, by 0.2 ± 0.01 (*p* = 0.0047; Table 2) than Northern Hemisphere species (Fig. 2). Moreover, there was an interaction between SMR and Arrhenius temperature (interaction coefficient −1.5 ± 0.4, *p* < 0.01; Table 2).

**Table 2:** Summary table for linear model for the relationship of metabolic scaling exponent, with mass-normalised standard metabolic rate (*SMR*), hemisphere and the interaction with Arrhenius temperature (temperature = 1,000/°K), where Northern Hemisphere is the reference (*Intercept*)

Coefficients	Estimate	SE	*t*-value	*p*-value
Intercept	−5.0	1.8	−2.8	0.017
SMR	5.2	1.6	3.3	0.0067
Arrhenius temperature	1.6	0.5	3.1	0.0086
Hemisphere S	0.2	0.1	3.5	0.0047
Arrhenius temperature × SMR	−1.5	0.4	−3.3	0.0066

The residual SE is 0.32 on 12 *df* and the multiple *R* ^2^-value is 0.66

### Mass-normalized SMR against Arrhenius temperature

**Fig. 3:**
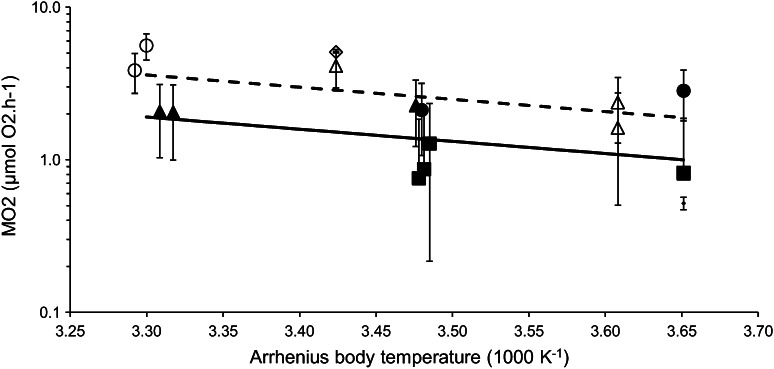
Calculated mass-normalized SMR (MO_2_ ± 1 SE, calculated for each species and normalised to 223 mg AFDM) against Arrhenius body temperature. Separate regression lines were fitted to Northern (*open symbols, dashed line*) and Southern (*closed symbols*) Hemisphere species, comprising 477 individuals across 17 species: bivalves of the genus *Laternula* (*circles*), gastropods of the family Buccinidae (*triangles*), echinoids of the infraorder Echinidae (*diamonds*), brachiopods (*squares*). Solutions fitted from results of a linear mixed effects model which accounted for the significant random effects of species nested in family. Note natural logarithm scale on the *y*-axis. Species tested at an Arrhenius body temperature of 3.48 K were slightly offset for clarity

Metabolic rate declined with a slope of −1.8 ± 0.7 with Arrhenius temperature, and increased linearly with temperature (Fig. 3; Table 3). Overall, Northern Hemisphere species had a higher metabolic rate for a given body temperature [by 0.6 Ln(SMR)]. The shallow gradient of this slope (1.8) gives a low *E*_a_ of 0.16 eV, which was significantly different (*Z* = 8, *p* < 0.01) to the average slope of −7.40 K from the MTE (Gillooly et al. 2001), and below the range of *E*_a_ predicted by the MTE (*E*_a_ = 0.2–1.2). *Q*_10_ comparisons between species within the taxonomic groups also showed the overall low sensitivity of SMR to temperature, with a median value of 1.4 and an interquartile range of 0.9–1.7, but with a high variability (ESM Table 2).

**Table 3:** Summary table for linear mixed effects model estimates fitted using restricted maximum likelihood for mass-normalised SMR {natural logarithm of mean respiration rate [ln(MO_2_)] of a standard-sized animal of 223 mg ash-free dry weight} as a function of the predictor, Arrhenius temperature (temperature = 1,000/°K), and the factor Hemisphere (Southern and Northern)

Model summary	AIC	logLik			
	445.6	−216.8			

Fixed effects	Value	SE	*t-*value	*p*-value	*df*
Reference	7.33	2.5	2.9	0.0036	405
Arrhenius temperature	−1.8	0.7	−3.2	0.031	9
Hemisphere S	−0.6	0.2	−4.1	0.011	9

Random effects	Family	Species	Residual		
	38.6 %	27.4 %	34.0 %		

The % variance of the random effects of species nested within family is reported. The reference is the Northern Hemisphere

*AIC* Akaike information criterion, *logLik* log likelihood

## Discussion

### Scaling of metabolic rate with body size

Here we provide evidence that metabolic scaling exponents are highly variable in a global sample of calcifying marine invertebrates. Overall, we found no greater support for a 2/3 versus a 3/4 AFDM scaling exponent of SMR. Although the 3/4 power scaling exponent between SMR and body mass is exhibited by a wide range of unicellular organisms, ectotherms and endotherms (Gillooly et al. 2001; Randall et al. 1997; West et al. 2003; Savage et al. 2004; West and Brown 2005), many studies have recently questioned the universality of the 3/4 power law and have found wide variation in the metabolic scaling exponent (Weibel et al. 2004; Weibel and Hoppeler 2005; Glazier 2010 and references therein). Mass-scaling exponents in the current study were mostly between 2/3 and 1 (*χ* ^2^ = 0.06, *p* = 0.80), fitting with the expectation of the metabolic-level boundaries hypothesis, which predicts variation in metabolic scaling exponents among taxa depending on the balance between surface area restrictions and the energy requirements of activity (Glazier 2010). The MLB hypothesis also predicts that metabolic scaling exponents should vary with temperature (Killen et al. 2010) implying a role for geographic environmental variation in explaining differences in metabolic rate among taxa, but also a significant difference between hemispheres.

One contributing factor to the variability in metabolic scaling exponents among species is the challenge of standardising metabolic rate for animals of different size. Most studies comparing metabolic rates over a wide range of species use tissue wet mass to quantify relative body size [Gillooly et al. 2001; Clarke and Johnston 1999; Addo-Bediako et al. 2002; but see Peck and Conway (2000) who used tissue dry mass]. Wet and dry mass measure both biological and skeletal tissues, and because individual tissues can vary in hydration state and differ in the proportion of organic versus inorganic tissue, have the potential to add variation into cross-taxa comparisons of metabolic rate. Different taxa can have markedly different ratios of skeletal to biological material (Peck 1993) and, moreover, at a global scale, this can vary with latitude, temperature and calcium saturation state (e.g. Watson et al. 2012). AFDM, which only includes the organic mass of biologically active tissue, is therefore likely to give better comparisons across distantly related taxa, and across environments. In the current study, the two scaling exponents below 0.66 were buccinids whose bodies are almost entirely enclosed in shells which may alter the geometry of flux of resources, particularly to the part of the animal that is permanently inside the shell.

### Comparisons of mass-corrected metabolic rate across taxa

Metabolic rate generally increased with temperature. The Antarctic bivalve *Laternula elliptica* and the Antarctic gastropod *Neobuccinum eatoni* had the lowest metabolic rate within their respective taxa. The SMR of *N. eatoni* at 0.7 °C (0.86 μmol O_2_ h^−1^ for a 1.7 g tissue dry mass individual; ESM Table 3) was lower than the metabolic rate found for the Antarctic *Trophon longstaffi* at 0.0 °C (1.44 μmol O_2_ h^−1^ for a 1.7 g tissue dry mass individual; Harper and Peck 2003), which was the previous lowest recorded metabolic rate of any marine gastropod. Metabolic rates from this carefully controlled comparison were broadly in agreement with values from the literature (ESM Table 3) but with variation of an order of magnitude as would be expected due to ecological and methodological differences between studies conducted at different locations. The energetic costs of producing and maintaining different somatic tissues and transferring energy in and out of storage compounds change seasonally and through ontogeny (Kooijman 2010) which will increase the variation in *E*_a_ (Gillooly et al. 2001). To reduce potential sources of variation, metabolic rate for most species in the current study was measured in summer and all individuals were starved for a period to reduce the variability due to recent feeding history.

The MTE encompasses a huge range of *E*_a_ values, 0.2–1.2 eV (Gillooly et al. 2001; although see Brown et al. 2004), which corresponds to a much wider range of *Q*_10_ values (1.33–5.72 over 10–30 °C) than normally measured for biological processes (Clarke 1983). Despite this, the temperature sensitivity (*E*_a_ of 0.16 eV) for the 17 species in the current study, calculated across a global seawater temperature range, was lower than predicted by the MTE; which is equivalent to a *Q*_10_ of 1.3. In a global analysis, Clarke and Johnston (1999) and Clarke (2004) found that fish species have a lower temperature dependence of mass-corrected metabolic rate than the average *E*_a_ of 0.65 eV predicted by the MTE (Gillooly et al. 2001). However, the difference reported by Clarke and Johnston (1999) and Clarke (2004) was much smaller than was found in the current study and was also disputed by a reanalysis of this data set (Gillooly et al. 2006). In a recent meta-analysis of rate/temperature relationships, the temperature sensitivity of a wide variety of traits across taxa had a median *E*_a_ of 0.55 eV (Dell et al. 2011; Huey and Kingsolver 2011), indicating that the distribution is skewed and more species have *E*_a_ values lower than the mean value (0.65 eV). Isolated tissues can have reduced temperature dependence compared to whole-animal active metabolic rate (Newell 1966, 1969) suggesting that variability in temperature sensitivity between species might, in part, be driven by ecological differences in relative energy allocation to different organs. Life history stage will therefore likely be responsible for some of the variation in temperature sensitivity in the literature, with differences in temperature sensitivity of SMR between juveniles and post-spawning individuals, compared to those with developing gonads (Bayne et al. 1976).

Some of the variation in *E*_a_ can be attributed to the different selective pressures acting on ecological traits in different species, for example, the increased low temperature performance of prey species compared to their predators (the life-dinner principle; Dell et al. 2011). Large departures of *E*a may also result from evolutionary trade-offs (Clarke 2004; Clarke and Fraser 2004) and non-climatic adaptations (Marshall and McQuaid 2011; Marshall et al. 2011a). One of the best-known examples is the eulittoral tropical gastropod, *Echinolittorina malaccana*, which enters metabolic depression at temperatures above 35 °C as an energy-saving strategy during periods of extreme high temperatures (Marshall and McQuaid 2011; Marshall et al. 2011a). This “non-climatic thermal adaptation” is an evolutionary trade-off (sensu Clarke and Fraser 2004) to save energy during prolonged periods of aestivation, when snails are not feeding, rather than a direct response to environmental temperature (Marshall et al. 2011b).

The lower metabolic scaling exponents and higher metabolic rates in Northern, compared to Southern, Hemisphere species indicate geographic differences in the factors controlling metabolic rate. An evolutionary trade-off that could explain these differences is metabolic cold adaptation (after Krogh 1914; Scholander et al. 1953). Marine invertebrates that experience a wide environmental temperature range across their distribution tend to have wider thermal tolerance windows (=eurythermal) (e.g. Sunday et al. 2011). Eurythermal species may require elevated SMR (e.g. Pörtner et al. 2007) to cope at the polar or subpolar ends of their distribution, resulting in extra physiological costs. Similarly, ectotherms living at constant low temperatures were thought to require higher resting metabolic rates, such as occur in polar environments, to overcome the rate-limiting effect of temperature. This may be true for species that have recently extended their range from warmer latitudes (e.g. range extension after the last ice age); however, many Antarctic marine fish and invertebrates which have evolved in very stable cold temperatures, have limited thermal tolerance windows (=stenothermal), reduced aerobic scopes, and subsequently low metabolic costs (Peck et al. 2006). Whilst there was evidence for MCA in insects (Addo-Bediako et al. 2002; although see Pörtner et al. 2007) and in boreal, but not Arctic, amphipods (Rastrick and Whiteley 2011), broad-scale latitudinal comparisons including polar species of fish (Holeton 1974; Steffensen 2002; Clarke and Johnston 1999 but see White et al. 2012) and bivalve molluscs (James et al. 1992), showed no signs of MCA. While here we only measured SMR of a few Northern Hemisphere species, there were clear differences between Northern and Southern Hemisphere species. The higher metabolic rate of northern species, but similar metabolic scaling exponent, suggests that evolutionary trade-offs may lead to different responses to climate warming between Northern and Southern Hemisphere species. These differences may have important effects on global predictions of metabolic rate and energy budgets into the future.

## Electronic supplementary material

Below is the link to the electronic supplementary material.

Supplementary material 1 (DOC 141 kb)
