# Supplementary material for: Low global sensitivity of metabolic rate to temperature in calcified marine invertebrates
Source: Oecologia. 2013 Sep 14;174(1):45–54. doi: 10.1007/s00442-013-2767-8 (PMC3884134; doi:10.1007/s00442-013-2767-8)
Supplement: Supplementary file 1 — Supplementary material 1 (DOC 141 kb) [file 442_2013_2767_MOESM1_ESM.doc]

Low global sensitivity of metabolic rate to temperature in calcified marine invertebrates

Sue-Ann Watson^a,1^, Simon A. Morley^b*^, Amanda E. Bates ^c^, Melody S. Clark^b^, Robert W. Day^d^, Miles Lamare^e^, Stephanie M. Martin^f^, Paul C. Southgate^g^, Koh Siang Tan^h^, Paul A. Tyler^a^, Lloyd S. Peck^b^

^a^ School of Ocean and Earth Science, University of Southampton, National Oceanography Centre Southampton, European Way, Southampton, SO14 3ZH, United Kingdom

^b^ British Antarctic Survey, Natural Environment Research Council, High Cross, Madingley Road, Cambridge, CB3 0ET, United Kingdom

^c^ Institute of Marine and Antarctic Studies, University of Tasmania, Hobart, Tasmania, Australia

^d^ Zoology Department, University of Melbourne, Parkville, 3010, Australia

^e^ Department of Marine Science, University of Otago, Dunedin, New Zealand

^f^ 14 Duck Lane, Eynesbury, St. Neots, Cambridgeshire, PE19 2DD, United Kingdom

^g^ Centre for Sustainable Tropical Fisheries and Aquaculture, School of Marine and Tropical Biology, James Cook University, Townsville, Queensland, 4811, Australia

^h^ Tropical Marine Science Institute, National University of Singapore, 14 Kent Ridge Road, Singapore 119223, Singapore

^1^ Present address: Australian Research Council Centre of Excellence for Coral Reef Studies and School of Marine and Tropical Biology, James Cook University, Townsville, Queensland, 4811, Australia

* Corresponding author e-mail: smor@bas.ac.uk

ESM Table 1. One sample Z tests of whether individual species scaling coefficients are significantly different from 0.66, 0.75 and 1.0. Accepted significance levels were Bonferroni corrected to p<0.016 and p<0.003 to account for the 3 comparisons.

|  |  |  |  | Test against 0.66 | | Test against 0.75 | | Test against 1.0 | |
| --- | --- | --- | --- | --- | --- | --- | --- | --- | --- |
| Taxon | Species | Scaling exponent | 95% CI | Z | P | Z | p | Z | P |
| Laternulid bivalves | *Laternula truncate* | 0.83 | 0.17 | 0.97 | 0.33 | 0.46 | 0.65 | 0.97 | 0.33 |
|  | *Laternula boschasina* | 0.91 | 0.94 | 0.57 | 0.57 | 0.36 | 0.72 | 0.20 | 0.84 |
|  | *Laternula recta* | 0.83 | 0.17 | 2.3 | 0.02 | 1.1 | 0.27 | 2.2 | 0.03 |
|  | *Laternula elliptica* | 0.84 | 0.08 | 4.8 | <0.003 | 2.4 | 0.02 | 4.4 | <0.003 |
| Buccinid gastropods | *Phos senticosus* | 0.66 | 0.31 | 0.02 | 0.99 | 0.56 | 0.58 | 2.2 | 0.03 |
|  | *Cantharus fumosus* | 0.57 | 0.26 | 0.67 | 0.50 | 1.3 | 0.18 | 3.2 | <0.003 |
|  | *Cominella lineolata* | 0.35 | 0.24 | 2.5 | <0.016 | 3.3 | <0.003 | 5.3 | <0.003 |
|  | *Buccinum undatum* | 0.75 | 0.17 | 0.98 | 0.33 | 0.05 | 0.96 | 2.9 | <0.003 |
|  | *Neobuccinum eatoni* | 0.92 | 0.20 | 2.5 | <0.016 | 1.6 | 0.10 | 0.83 | 0.40 |
|  | *Buccinum cf groenlandicum* | 0.38 | 0.22 | 2.5 | <0.016 | 7.4 | <0.003 | 5.6 | <0.003 |
|  | *Buccinum glaciale* | 0.59 | 0.10 | 1.4 | 0.15 | 3.2 | <0.003 | 8.1 | <0.003 |
| Echinoids | *Psammechinus miliaris* | 0.74 | 0.10 | 1.4 | 0.15 | 0.16 | 0.87 | 4.6 | <0.003 |
|  | *Sterechinus neumayeri* | 1.02 | 0.10 | 8.3 | <0.003 | 6.2 | <0.003 | 0.37 | 0.71 |
| Brachiopods | *Liothyrella neozelanica* | 0.72 | 0.19 | 0.70 | 0.48 | 0.28 | 0.80 | 3.0 | 0.003 |
|  | *Terebratella*  *sanguinea* | 0.73 | 0.16 | 0.86 | 0.39 | 0.32 | 0.75 | 3.6 | <0.003 |
|  | *Notosaria nigricans* | 0.37 | 0.49 | 1.6 | 0.10 | 1.67 | 0.10 | 2.7 | <0.016 |
|  | *Liothyrella uva* | 0.76 | 0.18 | 1.3 | 0.19 | 0.10 | 0.93 | 2.8 | <0.016 |

ESM Table 2 Q_10_ of standard metabolic rate of a standard sized animal (223 mg AFDM) within each taxa at temperatures (fig.2) for which standard metabolic rate was measured.

| **Species at upper temp.** | **Upper temp. (°C)** | **Species at lower temp.** | **Lower temp. (°C)** | **Q_10_** |
| --- | --- | --- | --- | --- |
| **Laternulid bivalves** |  |  |  |  |
| *Laternula truncata* | 29.9 | *Laternula recta* | 14.2 | 1.65 |
| *Laternula truncata* | 29.9 | *Laternula elliptica* | 0.7 | 1.53 |
| *Laternula boschasina* | 29.9 | *Laternula recta* | 14.2 | 1.66 |
| *Laternula boschasina* | 29.9 | *Laternula elliptica* | 0.7 | 1.53 |
| *Laternula recta* | 14.2 | *Laternula elliptica* | 0.7 | 1.40 |
| **Buccinid gastropods** |  |  |  |  |
| *Cantharus fumosus* | 28.3 | *Buccinum undatum* | 18.9 | 0.48 |
| *Cantharus fumosus* | 28.3 | *Cominella lineolata* | 14.2 | 0.80 |
| *Cantharus fumosus* | 28.3 | *Buccinum* cf. *Groenlandicum* | 4.0 | 0.98 |
| *Cantharus fumosus* | 28.3 | *Buccinum glaciale* | 4.0 | 0.85 |
| *Cantharus fumosus* | 28.3 | *Neobuccinum eatoni* | 0.7 | 1.62 |
| *Phos senticosus* | 28.3 | *Buccinum undatum* | 18.9 | 0.53 |
| *Phos senticosus* | 28.3 | *Cominella lineolata* | 14.2 | 0.86 |
| *Phos senticosus* | 28.3 | *Buccinum* cf. *Groenlandicum* | 4.0 | 1.02 |
| *Phos senticosus* | 28.3 | *Buccinum glaciale* | 4.0 | 0.88 |
| *Phos senticosus* | 28.3 | *Neobuccinum eatoni* | 0.7 | 1.68 |
| *Buccinum undatum* | 18.9 | *Cominella lineolata* | 14.2 | 2.30 |
| *Buccinum undatum* | 18.9 | *Buccinum* cf. *Groenlandicum* | 4.0 | 1.54 |
| *Buccinum undatum* | 18.9 | *Buccinum glaciale* | 4.0 | 1.23 |
| *Buccinum undatum* | 18.9 | *Neobuccinum eatoni* | 0.7 | 3.06 |
| *Cominella lineolata* | 14.2 | *Buccinum* cf. *Groenlandicum* | 4.0 | 1.28 |
| *Cominella lineolata* | 14.2 | *Buccinum glaciale* | 4.0 | 0.92 |
| *Cominella lineolata* | 14.2 | *Neobuccinum eatoni* | 0.7 | 3.37 |
| *Buccinum* cf. *Groenlandicum* | 4.0 | *Neobuccinum eatoni* | 0.7 | 70.21 |
| *Buccinum glaciale* | 4.0 | *Neobuccinum eatoni* | 0.7 | 200.01 |
| **Echinoids** |  |  |  |  |
| *Psammechinus miliaris* | 18.9 | *Sterechinus neumayeri* | 0.7 | 4.65 |
| **Brachiopods** |  |  |  |  |
| *Liothyrella neozelanica* | 14.1 | *Liothyrella uva* | 0.7 | 1.37 |
| *Terebratella sanguinea* | 14.1 | *Liothyrella uva* | 0.7 | 0.84 |
| *Notosaria nigricans* | 14.1 | *Liothyrella uva* | 0.7 | 0.56 |

ESM Table 3. Summary of rates of oxygen consumption measured in the current study with data for the same species from the literature. Where possible units were converted to µmol O_2_.h^-1^ according to Peck *et al*. (1986b). Animals were held in the laboratory aquarium with no food provided other than that available in the inflowing seawater.

| **Species** | **Temp. (ºC) (mean ± 1 s.d.)** | **Respiration rate (mean ± 1 s.d.)** | **Pre-treatment** | **Animal size** | **Source** |
| --- | --- | --- | --- | --- | --- |
| **Laternulid Bivalves** |  |  |  |  |  |
| *Laternula truncata* | 29.9 (± 0.5) | 6.03 (± 2.60) µmol O_2_.h^-1^ | Fasted for 3 days | 40.0 mm shell length (223 mg) | Current study |
| *Laternula boschasina* | 29.9 (± 0.5) | 6.05 (± 3.00) µmol O_2_.h^-1^ | Fasted for 3 days | 33.8 mm shell length (223 mg) | Current study |
| *Laternula recta* | 14.2 (± 0.1) | 2.74 (± 0.45) µmol O_2_.h^-1^ | Fasted for 7 days | 41.5 mm shell length (223 mg) | Current study |
| *Laternula elliptica* | 0.7 (± 0.2) | 1.75 (± 0.37) µmol O_2_.h^-1^ | Fasted for 14 days | 21.9 mm shell length (223 mg) | Current study |
| *Laternula elliptica* | 0.5 | 12.9 µmol O_2_.h^-1^ | Fasted for 2 days | 60 mm shell length | Ahn and Shim (1998) |
| *Laternula elliptica* | 0.0 | 134 µmol O_2_.h^-1^ | Fasted for 14 days | 12.5 mg | [30] |
| *Laternula elliptica* | Winter (Aug)  Summer (Mar) | 4.3 µmol O_2._h^-1^  12.9 µmol O_2._h^-1^ | Held for 4 days in an aquarium | 50 mm shell length | Brockington (2001) |
| *Laternula elliptica* | -0.4 (Feb) | 19.2 µmol O_2._h^-1^ | Held for 1–5 days in an aquarium | 50 mm shell length | [33] |
| **Buccinid Gastropods** |  |  |  |  |  |
| *Cantharus fumosus* | 28.3 (± 0.1) | 2.01 (±0.58) µmol O_2._h^-1^ | Fasted for 5 days | 27.4 mm shell height (223 mg) | Current study |
| *Phos senticosus* | 28.3 (± 0.1) | 2.20 (± 0.60) µmol O_2._h^-1^ | Fasted for 5 days | 31.7 mm shell height (223 mg) | Current study |
| *Cominella lineolata* | 14.2 (± 0.1) | 2.73 (± 1.70) µmol O_2._h^-1^ | Fasted for 7 days | 25.5 mm shell height (223 mg) | Current study |
| *Buccinum undatum* | 18.9 (± 0.4) | 4.04 (± 1.12) µmol O_2._h^-1^ | Fasted for 7 days | 27.3 mm shell height (223 mg) | Current study |
| *Buccinum undatum* | 7.5 (Mar),  10.5 (May)  15 (Aug) | 2.6-122 µmol O_2_.h^-1^  14-191 µmol O_2_.h^-1^  9.7-188 µmol O_2_.h^-1^ | Non-fasted | 30 to ≥ 90 mm shell length (= shell height) | Kideys (1998) |
| *Neobuccinum eatoni* | 0.7 (± 0.2) | 0.53 (± 0.12) µmol O_2._h^-1^ | Fasted for 14 days | 20.9 mm shell height (223 mg) | Current study |
| *Buccinum* cf. *groenlandicum* | 4 | 2.12 (± 0.84) µmol O_2._h^-1^ | Fasted for 12 days | 26.1 mm shell height (223 mg) | Current study |
| *Buccinum glaciale* | 4 | 2.98 (± 0.96) µmol O_2._h^-1^ | Fasted for 12 days | 28.2 mm shell height (223 mg) | Current study |
| **Echinoids** |  |  |  |  |  |
| *Psammechinus miliaris* | 18.9 (± 0.4) | 5.32 (± 1.75) µmol O_2._h^-1^ | Fasted for 7 days | 20.9 mm test diameter (223 mg) | Current study |
| *Psammechinus miliaris* | 9-14 | 0.47 µmol O_2_.h^-1^.g^-1^ (wet weight) for 29-37 mm test diameter echinoids | Fasted for 2 weeks | dry mass specific | Otero-Villanueva *et al*. (2004) |
| *Sterechinus neumayeri* | 0.7 (± 0.2) | 0.33 (± 0.08) µmol O_2._h^-1^ | Fasted for 14 days | 14.6 mm test diameter (223 mg) | Current study |
| *Sterechinus neumayeri* | -1.8 (winter)  1.2 (summer) | 0.33–0.65 µmol O_2._h^-1^ (between 2 sites)  1.44–1.62 µmol O_2._h^-1^ | Non-fasted | 24.4 mm test diameter | Brockington & Peck (2001) |
| **Brachiopods** |  |  |  |  |  |
| *Liothyrella neozelanica* | 14.1 (± 0.8) | 1.45 (± 0.52) µmol O_2._h^-1^ | Fasted for 9 days | 50.2 (from regression) mm pedicle valve length (223 mg) | Current study |
| *Terebratella sanguinea* | 14.1 (± 0.8) | 0.76 (± 0.33) µmol O_2._h^-1^ | Fasted for 9 days | 223 mg | Current study |
| *Terebratella sanguinea* | 10 | 30.3 µmol O_2_.h^-1^.g^-1^ (dry tissue mass) | Held for ≥ 7 days in an aquarium | dry mass specific | Shumway (1982) |
| *Notosaria nigricans* | 14.1 (± 0.8) | 0.45 (± 0.20) µmol O_2._h^-1^ | Fasted for 9 days | 223 mg | Current study |
| *Liothyrella uva* | 0.7 (± 0.2) | 0.96 (± 0.49) µmol O_2._h^-1^ | Fasted for 21 days | 33.2 (from regression) mm pedicle valve length (223 mg) | Current study |
| *Liothyrella uva* | 0.0 | 0.24 µmol O_2_.h^-1^ | Fasted for 4-6 weeks | 35 mm length  (210 mg AFDM) | Peck *et al*. (1986b) |
| *Liothyrella uva notorcadensis* | 0.0 | 0.240 µmol O_2_.h^-1^ | Fasted for 2 weeks | 35 mm length  (238 mg AFDM) | Peck *et al*. (1986a) |
| *Liothyrella uva* | 0.0 (summer) | 0.103 µmol O_2_.h^-1^ | Non-fasted | 50 mg AFDM | Peck *et al*. (1987) |
| *Liothyrella uva* | 0.0 (summer) | 0.45 – 0.67 µmol O_2_.h^-1^ | Fasted for up to 2 weeks | 286 mg AFDM  (38.6 mm length) | Peck (1989) |
| *Liothyrella uva* | 0.76 (summer) | 0.64 µmol O_2_.h^-1^ (fasted for 25 days)  0.97 – 1.06 µmol O_2_.h^-1^ (after feeding) | All stages | 290 mg AFDM | Peck (1996) |
| *Liothyrella uva* | 0.5 (summer) | 0.0422 µmol O_2_.h^-1^ | Fasted for 35 days | 50 mg AFDM | Peck *et al*.(1997) |

Ahn IY, Shim JH (1998) Summer metabolism of the Antarctic clam, *Laternula elliptica* (King and Broderip) in Maxwell Bay, King George Island and its implications. J Exp Mar Biol Ecol 224: 253-264

Brockington S (2001) The seasonal energetics of the Antarctic bivalve *Laternula elliptica* (King and Broderip) at Rothera Point, Adelaide Island. Polar Biol 24: 523-530

Brockington S, Peck LS (2001) Seasonality of respiration and ammonium excretion in the Antarctic echinoid *Sterechinus neumayeri*. Mar Ecol Prog Ser 219: 159-168

Kideys AE, (1998) Physiological energetics of *Buccinum undatum* L. (Gastropoda) off Douglas, Isle of Man (the Irish Sea). Turk J Zool 22: 49-61

Otero-Villanueva MM, Kelly MS, Burnell G (2004) How diet influences energy partitioning in the regular echinoid *Psammechinus miliaris*; constructing an energy budget. J Exp Mar Biol Ecol 304: 159-181

Peck LS (1989) Temperature and basal metabolism in two Antarctic marine herbivores. J Exp Mar Biol Ecol 127: 1-12

Peck LS (1996) Metabolism and feeding in the Antarctic brachiopod *Liothyrella uva*: A low energy lifestyle species with restricted metabolic scope. Proc Roy Soc B 263: 223-228

Peck LS, Morris DJ, Clarke A (1986a) Oxygen consumption and the role of caeca in the Recent Antarctic brachiopod *Liothyrella uva notorcadensis* (Jackson, 1912). In: Racheboeuf PR and Emig CC (eds.) Les Brachiopodes fossiles et actuels. Biostratigraphie du Paléozoique, 4: 500 pp

Peck LS, Morris DJ, Clarke A, Holmes LJ (1986b) Oxygen-consumption and nitrogen-excretion in the Antarctic brachiopod *Liothyrella uva* (Jackson, 1912) under simulated winter conditions. J Exp Mar Bio Ecol 104: 203-213

Peck LS, Clarke A, Holmes LJ (1987) Summer metabolism and seasonal changes in biochemical composition of the Antarctic brachiopod *Liothyrella uva* (Broderip, 1833). J Exp Mar Biol Ecol 114: 85-97

Peck LS, Brockington S, Brey T (1997) Growth and metabolism in the Antarctic brachiopod *Liothyrella uva*. Philos T R Soc B 352: 851-858

Shumway S E (1982) Oxygen consumption in brachiopods and the possible role of punctae. J Exp Mar Biol Ecol 58: 207-220
